# Deficiency of Mitochondrial Functions and Peroxidation of Frontoparietal Cortex Enhance Isoflurane Sensitivity in Aging Mice

**DOI:** 10.3389/fnagi.2020.583542

**Published:** 2020-12-03

**Authors:** Cansheng Gong, Donghang Zhang, Wei Ou, Mengchan Ou, Peng Liang, Daqing Liao, Weiyi Zhang, Tao Zhu, Jin Liu, Cheng Zhou

**Affiliations:** ^1^Laboratory of Anesthesia & Critical Care Medicine, Translational Neuroscience Center, National Clinical Research Center for Geriatrics, West China Hospital of Sichuan University, Chengdu, China; ^2^Department of Anesthesiology, West China Hospital of Sichuan University, Chengdu, China; ^3^Department of Anesthesiology, Shengli Clinical Medical College of Fujian Medical University, Fujian Provincial Hospital, Fuzhou, China

**Keywords:** anesthetics sensitivity, isoflurane, oxidation, aging, mitochondrial bioenergetics

## Abstract

**Background**: Hypersensitivity to general anesthetics may predict poor postoperative outcomes, especially among the older subjects. Therefore, it is essential to elucidate the mechanism underlying hypersensitivity to volatile anesthetics in the aging population. Given the fact that isoflurane sensitivity increases with aging, we hypothesized that deficiencies of mitochondrial function and elevated oxidative levels in the frontoparietal cortex may contribute to the enhanced sensitivity to isoflurane in aging mice.

**Methods**: Isoflurane sensitivity in aging mice was determined by the concentration of isoflurane that is required for loss of righting reflex (LORR). Mitochondrial bioenergetics of the frontoparietal cortex was measured using a Seahorse XFp analyzer. Protein oxidation and lipid oxidation in the frontoparietal cortex were assessed using the Oxyblot protein oxidation detection kit and thiobarbituric acid reactive substance (TBARS) assay, respectively. Contributions of mitochondrial complex II inhibition by malonate and peroxidation by ozone to isoflurane sensitivity were tested *in vivo*. Besides, effects of antioxidative therapy on mitochondrial function and isoflurane sensitivity in mice were also measured.

**Results**: The mean concentration of isoflurane that is required for LORR in aging mice (14–16 months old) was 0.83% ± 0.13% (mean ± SD, *n* = 80). Then, the mice were divided into three groups as sensitive group (S group, mean − SD), medium group (M group), and resistant group (R group, mean + SD) based on individual concentrations of isoflurane required for LORR. Activities of mitochondrial complex II and complex IV in mice of the S group were significantly lower than those of the R group, while frontoparietal cortical malondialdehyde (MDA) levels were higher in the mice of S group. Both inhibition of mitochondrial complexes and peroxidation significantly decreased the concentration of isoflurane that is required for LORR *in vivo*. After treatment with idebenone, the levels of lipid oxidation were alleviated and mitochondrial function was restored in aging mice. The concentration of isoflurane that required for LORR was also elevated after idebenone treatment.

**Conclusions**: Decreased mitochondrial functions and higher oxidative stress levels in the frontoparietal cortex may contribute to the hypersensitivity to isoflurane in aging mice.

## Introduction

The mechanism of how general anesthetics work is not fully clear (Kennedy, 2005). Volatile anesthetics are widely used in clinical settings, and the mechanism of their action is relatively complex because volatile anesthetics such as isoflurane and sevoflurane can interact with multiple molecular targets (Campagna et al., 2003; Sonner et al., 2003; Forman and Chin, 2008).

The sensitivity to volatile anesthetics increases with aging in both patients and laboratory animals (Magnusson et al., 2000; Van Cleve et al., 2015). The MAC (minimum alveolar concentration) of volatile anesthetics decreases in the elderly (Chemali et al., 2015). Altered sensitivity to volatile anesthetics can affect the depth of general anesthesia during surgery. Several studies indicate that the depth of general anesthesia may predict postoperative outcomes (Sessler et al., 2012; Willingham et al., 2015). Cumulative deep hypnosis and prolonged burst suppression during general anesthesia are significant predictors of postoperative mortality (Monk et al., 2005; Watson et al., 2008; Kertai et al., 2010). Postoperative mortality is higher in the patients with combined lower MAC fraction and lower BIS (bispectral index), which stands for hypersensitivity to volatile anesthetics and deep sedation, respectively (Sessler et al., 2012). The patients with electroencephalogram suppression at lower concentrations of volatile anesthetics are susceptible to postoperative delirium (Sieber et al., 2010; Radtke et al., 2013; Fritz et al., 2016, 2018). Therefore, it is essential to elucidate the mechanism underlying hypersensitivity to volatile anesthetics in the aging population.

Mitochondria are the center of various cellular process including ATP production, reactive oxygen species generation, and intracellular Ca^2+^ signaling. Neurons critically depend on mitochondrial function to establish membrane excitability and to execute the complex processes of neurotransmission and plasticity (Kann and Kovacs, 2007). Mitochondrial function, especially mitochondrial bioenergetics, contributes to the sensitivity to volatile anesthetics (Zimin et al., 2016, 2018). Defects of mitochondrial complex-dependent oxidative phosphorylation capacity can increase sensitivity to volatile anesthetics, which have been found in various animal models, including *Caenorhabditis elegans* (Kayser et al., 1999, 2004), mice (Quintana et al., 2012; Carspecken et al., 2018), and human beings (Morgan et al., 2002). Further study showed that the mitochondrial deficiency impaired synaptic ATP production and excitatory vesicle endocytosis/exocytosis, leading to the hypersensitivity to isoflurane (Zimin et al., 2018). Besides, oxidative stress compromises neuronal excitability and the capacity of generating of action potentials (Pardillo-Diaz et al., 2015) and is also able to enhance the sedative effects of diazepam. Since elevated levels of oxidative stress, defects of mitochondrial function, and metabolic impairments are common changes along with aging (Grimm and Eckert, 2017; Ximerakis et al., 2019), we test the hypothesis in this present study that the mitochondrial function, especially bioenergetics, and the levels of oxidative stress in brain tissue contribute to the different sensitivity to the volatile anesthetic isoflurane in aging mice.

## Materials and Methods

### Animals

The Institutional Animal Care and Use Committee of West China Hospital (Sichuan University, Chengdu, China) reviewed and approved all the protocols which adhere to the applicable ARRIVE guidelines. Eighty male C57BL/6J mice (14–16 months old, 31.4 ± 2.3 g) were used for assessing isoflurane sensitivity and mitochondrial functions. Young male C57BL/6J mice (2–3 months old) were used for assessing the effects of inhibition of mitochondrial functions and peroxidation on isoflurane sensitivity *in vivo*. All the mice were housed in standard conditions with free access to food and water and were maintained on a 12-h light dark cycle.

### Behavioral Test of Loss of Righting Reflex (LORR) in Mice

An open-circuit anesthetizing system was used to measure isoflurane-induced LORR in mice. This system contained six isolated cylindrical chambers with an internal diameter of 10 cm and a length of 15 cm. The system was capable of holding six mice simultaneously. A heating pad was placed under the chamber to maintain the body temperature of mice at 36 ± 0.5°C. The carrier gas was 100% O_2_, and the gas flow was 2.0 l/min. Outlet concentrations of isoflurane (RWD Life Science, Shenzhen, China), O_2_, and CO_2_ were continuously monitored by a gas analyzer (Philips HP M1026B). For each trial, the concentration of isoflurane was increased from 0.50% until 1.15% with an increment of 0.05% (13 concentrations in total). Each concentration of isoflurane was maintained for 15 min, then the cylindrical chambers were rotated. LORR was defined as the mouse was unable to turn itself prone onto all feet within 30 s. The minimum concentration of isoflurane that is required for LORR in individual mice was recorded as MAC_LORR_. Based on MAC_LORR_ of each mouse, the mice were divided into sensitive group (S group), medium group (M group), and resistant group (R group). The dividing lines were mean − standard deviation and mean + standard deviation of the MAC_LORR_. If MAC_LORR_ of the mice was lower than the mean − standard deviation, it was divided into sensitive group (S), and if the MAC_LORR_ of the mice was higher than mean + standard deviation, it was divided into resistant group (R), while the mice with MAC_LORR_ between mean − standard deviation and mean + standard deviation were into medium group (M).

### Exhaustive Swimming Test

After the measurement of MAC_LORR_ of isoflurane, exhaustive swimming test was employed to assess fatigue tolerance of the mice. The exhaustive swimming test was carried out in a swimming tank (40 × 50 × 40 cm^3^) filled with warm water (30 ± 1°C). Lead pieces equaled to 5% of body weight were attached to the tail of mouse ~1 cm distal from the body. Then, the mouse was put into the swimming tank. The mouse was considered to be exhausted when it failed to rise to the surface of the water for 10 s.

### Morris Water Maze Test

Spatial learning and memory of mice were assessed by Morris water maze as described previously (Vorhees and Williams, 2006). The water maze was a round pool with diameter of 90 cm and depth of 50 cm. The pool was divided into four quadrants with a separate entry point in each quadrant. Different-shaped objects were attached to the wall of pool to provide spatial visual clues. The water temperature was kept at 30 ± 1°C. A circular platform (with diameter of 6 cm) was placed 1 cm below the water surface. The quadrant with the circular platform was defined as the target quadrant, and the platform was 30 cm away from the wall of pool. An automatic video camera was used to record the swimming routes of mice. During the orientation navigation experiment, every mouse was trained three times a day for consecutive 4 days. For each training trial, the mouse was gently placed into the water facing the wall of pool. If the mouse found the platform within 90 s, it was allowed to stay on the platform for 15 s and the time period was recorded as escape latency. Otherwise, it would be guided to the platform to stay for 15 s, and the escape latency was recorded as 90 s. During the spatial probe test, the platform was removed and the mouse was allowed to swim free for 90 s. The area previously with the platform was defined as the target quadrant. The numbers of entrance into the target area and the time spent in the target quadrant and opposite quadrant as well as in the area around target were recorded. The software SMART (Panlab, Barcelona, Spain) was used to analyze the swimming trace of each mouse.

### Isolation of Brain Mitochondria

After the behavioral tests, frontoparietal cortical mitochondria were isolated as previously described (Pandya et al., 2007, 2013, 2014). Briefly, the brain of mice was rapidly removed and put into an ice-cold mitochondrial isolation buffer (MIB) composed of the following (in mM): 220 mannitol, 70 sucrose, 10 HEPES (N-2-hydroxyethylpiperazine-N′-2-ethane sulfonic acid), 1 EGTA (ethylene glycol tetraacetic acid), and 0.2% BSA (bovine serum albumin), pH = 7.2. The frontoparietal cortex was dissected and placed into a Teflon homogenizer containing 5-fold volumes of isolation buffer. The tissue was homogenized with an electric motor for eight times at a speed of 200 rpm and then centrifuged at 600× *g* for 5 min. The supernatant was transferred into a fresh tube and spun at 8,000× *g* for 10 min. The supernatant was discarded, and the pellet was resuspended in a 2-ml isolation buffer and centrifuged again at 8,000× *g* for 10 min. Then, the mitochondrial pellet was resuspended in mitochondrial storage buffer containing the following (in mM): 250 sucrose, 10 HEPES, 5 sodium succinate, 2 K_2_HPO_4_, 1 ATP, and 1 ADP. Protein concentrations were determined by using the BCA protein assay kit (Beyotime Biotechnology, Shanghai, China).

### Measurement of Isolated Mitochondria Respiration

The Seahorse Bioscience XFp extracellular flux analyzer (Seahorse Bioscience, North Billerica, MA, USA) was used to measure the respiration of acute isolated mitochondria (3 μg of mitochondrial protein per well). The coupling assay and electron flow assay were performed as previously described (Rogers et al., 2011; Subramaniam et al., 2014). In the coupling assay, basal respiration was driven by 10 mM succinate (Complex II respiration) in the presence of 2 μM rotenone. ADP at 4 mM, oligomycin at 3 μM, FCCP [carbonyl cyanide 4-(trifluoromethoxy)phenylhydrazone] at 4 μM, and antimycin A at 4 μM were sequentially injected to measure state 3_ADP_, state 4o, and state 3u, and to inhibit respiration, respectively. In the electron flow experiment, basal respiration was driven by 10 mM pyruvate, 2 mM malate, and 4 μM FCCP (uncoupled Complex I respiration). Rotenone at 2 μM, succinate at 10 mM, antimycin A at 4 μM, and ascorbate/TMPD (N, N, N′, N′-tetramethyl-p-phenylenediamine) at 10/0.1 mM were sequentially injected. The experiment initiated in an uncoupled state with substrates of Complex I. Sequentially, rotenone (Complex I inhibitor), succinate (Complex II substrate), antimycin A (Complex III inhibitor), and ascorbate/TMPD (electron donors to cytochrome C/Complex IV) were added. Thus, the experiment was feasible to determine Complex I, II, and IV-dependent respiration.

### Measurement of Oxidation Levels of Protein and Lipid in Brain

Frontoparietal cortex samples of mice were collected and analyzed for oxidation levels. Protein oxidation was determined by protein carbonylation using the OxyBlot protein oxidation detection analysis kit (Millipore, Billerica, MA, USA, S7150, lot: 3127842). The protocol was according to the instruction of the manufacturer. Briefly, sample protein 10 μg was denatured by adding 5 μl SDS (12%). Derivatization was achieved subsequently by adding 5 μl DNPH (10 mM) solution. After incubation for 15 min, samples were neutralized with 7.5 μl neutralization solution. Then, the samples were loaded on 12% polyacrylamide gels for electrophoretic separation and then transferred to a PVDF membrane. The membrane was washed by TBST solution containing 0.04% (v/v) Tween 20 (WB) followed by blocking with 5% milk before incubation with the rabbit polyclonal anti-DNP primary antibody (1:150; Rabbit Anti-DNP antibody, Millipore, Billerica, MA, USA, in Oxyblot kit, S7150, lot: 3127842) at 4°C overnight. Oxidized proteins were detected with secondary antibodies (1:300; Goat Anti-Rabbit IgG (HRP-conjugated), Millipore, Billerica, MA, USA, in Oxyblot kit, S7150, lot: 3127842) and chemiluminescence reagents (Millipore, Billerica, MA, USA). GAPDH (1:20,000; Proteintech, Wuhan, China, 10494-1-AP) was used as reference. Image J was used to assess pixel density of the resultant blots (Fragoulis et al., 2017; von Leden et al., 2017).

The concentration of malondialdehyde (MDA) in the mouse frontoparietal cortex was measured by the thiobarbituric acid reactive substance (TBARS) assay according to the manufacturer’s instructions (Jiancheng Bio-chemical Institute, Nanjing, China). The absorbance of samples was measured at a wavelength of 532 nm (Ma et al., 2016). The concentrations of superoxide dismutase (SOD) and glutathione (GSH) in mouse frontoparietal cortical homogenates were measured by the hydroxylamine method according to the manufacturer’s instructions (Jiancheng Bio-chemical Institute, Nanjing, China), and the absorbance was measured at a wavelength of 550 nm or 405 nm, respectively.

### Iron/Ascorbate-Induced Oxidation of Mitochondria

To explore the effects of peroxidation on mitochondrial respiration, ascorbic acid and Fe^2+^ were used to induce peroxidation as previously described (Cardoso et al., 1999; Brailovskaya et al., 2001). Frontoparietal cortical mitochondria from young mice (1 mg/ml of protein, final concentration) were incubated at 30°C for 15 min in the Na^+^ medium containing the following (in mM, pH = 7.4): 132 NaCl, 1 KCl, 1 MgCl_2_, 10 glucose, and 10 HEPES–Tris, with different concentrations of iron/ascorbate. A series of concentrations of iron/ascorbate were added to the medium as follows (in μM): 0/0 μM, 0.125/40 μM, 0.25/80 μM, 1.25/400 μM, or 2.5/800 μM Fe^2+^/ascorbic acid, respectively (Cardoso et al., 1999). Then, mitochondria were centrifuged (8,000× *g* for 10 min) and the pellets were washed and resuspended in Na^+^ medium for Seahorse XFp analysis (3 μg of mitochondria was required per well for each analysis, repeated three times for each sample).

### Malonate Treatment

To determine whether the inhibition of Complex II would affect the isoflurane sensitivity *in vivo*, 20 mice from the M group were randomly selected and preinjected (intraperitoneal) with malonate (375 mg/kg, CAS: 26522-85-0; Lu et al., 2018) or saline 30 min before isoflurane exposure. The MAC_LORR_ of isoflurane was then determined. To exclude the direct effect of malonate on locomotion, the locomotor activity was tested as previously described (Zhao et al., 2016). Briefly, a mice autonomic locomotor activity tester (Taimeng Tech Limited, Chengdu, China) was employed in this experiment. Mice were put into individual black chambers (15 × 15 × 10 cm^3^), which were placed in an recording system with infrared transmitters and receivers for recording horizontal and vertical movements for 30 min. Before testing, mice were put into the chambers for 1 h daily for 3 days to make them acclimate to the chambers.

### Ozone Exposure

To test the effect of peroxidation on isoflurane sensitivity *in vivo*, ozone was used to induce peroxidation in mice. Briefly, young mice (*n* = 12) were placed in a closed chamber connected to a variable flux ozone generator which continuously converts air into ozone. The mice were exposed to ozone (0.4 ppm) for 4 h daily for consecutive 7 days. An ozone monitor was used to determine the ozone concentration inside the chamber. Control mice (*n* = 8) were exposed to air.

To exclude the influence of ozone exposure on locomotion, an open-field test was used to measure the locomotor activity of mice. The open-field test was performed before the last exposure of ozone or air. Mice were placed in the center of the open field (60 × 25 × 45 cm^3^) to record the total distance and mean velocity during 10 min.

After the ozone exposure and behavioral tests, the mice were sacrificed and frontoparietal cortices were dissected for mitochondrial function (respiratory coupling experiment and electron flow experiment) and oxidative stress (MDA and protein carbonylation level) analyses.

### Idebenone Treatment

To test whether antioxidants can affect isoflurane sensitivity and mitochondrial function, 28 mice from the M group were randomly selected and treated with idebenone or vehicle (fish oil). Idebenone (100 mg/kg, CAS: 58186-27-9), an important antioxidant for the cell membrane and an elementary constituent of the ATP-producing mitochondrial electron transport chain, was suspended in fish oil and applied daily by oral gavage. Vehicle was administrated in the same volume in vehicle group. After treatment of idebenone for consecutive 60 days, sensitivity to isoflurane, frontoparietal cortical mitochondrial function (respiratory coupling experiment and electron flow experiment), and MDA and protein carbonylation levels were measured.

### Statistical Analyses

Quantitative data are present as mean ± standard deviation (SD). All statistical analyses were performed using SPSS 22.0 software (SPSS Inc., New York, NY, USA). Normality of the data distribution was assessed using the Shapiro–Wilk test. Navigation experiment performance and locomotor activity after malonate treatment were analyzed using a two-way analysis of variance (ANOVA) with repeated measures. The exhaustive swimming time, probe experiment performance, complex respiration, and MDA and protein carbonylation among the three groups were analyzed by a one-way ANOVA followed by the LSD *post hoc*. The locomotor activity tested by open field was analyzed by two-tailed unpaired student’s *t*-test. *P* < 0.05 was considered statistically significant.

## Results

### The MAC_LORR_ of Isoflurane Is Normally Distributed in Aging Mice

**Figure 1:**
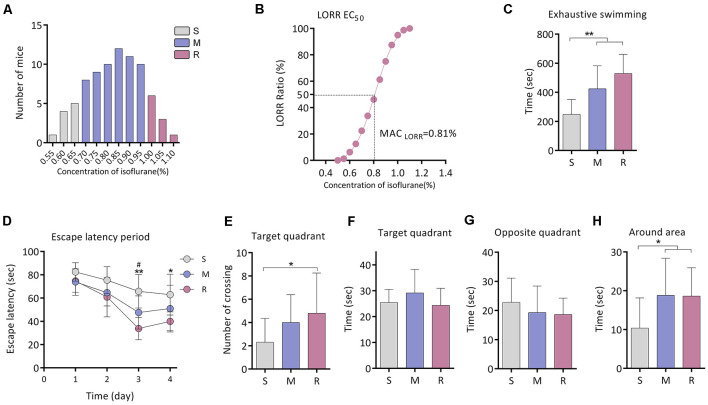
Exercise tolerance and performance in the Morris Water Maze (MWM) of aging mice with different isoflurane sensitivities. **(A)** Distribution of the individual isoflurane concentration required for LORR (*n* = 80). **(B)** EC_50 LORR_ of mice (*n* = 80). **(C)** Comparison of exhaustive swimming time among three groups with different isoflurane sensitivities (*n* = 10 for S and R groups, *n* = 15 for M group, **P < 0.01 vs. S group by one-way ANOVA). **(D)** Learning curve for the mean escape latency (*n* = 10 for S and R groups, *n* = 15 for M group, *P < 0.05 and **P < 0.01 vs. S group, and ^#^*P* < 0.05 vs. M group by two-way ANOVA with repeated measures). **(E)** Number of crossing the target area (*n* = 10 for S and R groups, *n* = 15 for M group, *P < 0.05 vs. S group by one-way ANOVA). **(F)** Time spent in the target quadrant (*n* = 10 for S and R groups, *n* = 15 for M group, *P* > 0.05 among the three groups by one-way ANOVA). **(G)** Time spent in the opposite quadrant (*n* = 10 for S and R groups, *n* = 15 for M group, *P* > 0.05 among the three groups by one-way ANOVA). **(H)** Time spent in the area around target (*n* = 10 for S and R groups, *n* = 15 for M group, *P < 0.05 vs. S group by one-way ANOVA). S, sensitive group; R, resistant group; M, medium group; LORR, loss of righting reflex. Data are presented as mean ± SD.

The individual minimum concentration of isoflurane required for LORR was statistically normally distributed (*n* = 80; Figure 1A). MAC_LORR_ of isoflurane in all the mice was 0.81 (0.80, 0.82) % (*n* = 80; Figure 1B). The mean and standard deviation were calculated as 0.83% ± 0.13%. According to the distribution, the mice were divided into sensitive group (S group, *n* = 10), medium group (M group, *n* = 60), and resistant group (R group, *n* = 10) group. The mean MAC_LORR_ for the S, M, and R groups was 0.62% ± 0.03%, 0.83% ± 0.08%, and 1.03% ± 0.04%, respectively. The mean MAC_LORR_ was significantly higher in the R group than that of the S group (*P* < 0.0001).

### Locomotivity, Spatial Learning, and Memory Are Better in R Group

Fifteen mice from the M group were randomly selected for this experiment part. The exhaustive swimming time of mice was significantly shorter in the S group (248.1 ± 102.5 s, *n* = 10) than that of the M group (424.3 ± 158.1 s, *n* = 15, *P* = 0.004 vs. S group) or R group (529.4 ± 132.6 s, *n* = 10, *P* < 0.001 vs. S group; Figure 1C).

In the Morris water maze test, significant differences in escape latency were detected between groups by two-way ANOVA (*P* < 0.0001; Figure 1D). During training, there was no difference among the groups on day 1. The escape latency was longer in the S group when compared with the R group on day 3 (65.8 ± 14.3 s vs. 33.9 ± 9.6 s, *n* = 10, *P* = 0.006; Figure 1D) and day 4 (62.9 ± 17.5 s vs. 40.0 ± 8.2 s, *n* = 10, *P* = 0.013; Figure 1D). During the spatial probe test, the times across the platform were decreased in the S group than that of the R group (2.3 ± 2.1 vs. 4.8 ± 3.5 times, *n* = 10, *P* = 0.043; Figure 1E). No significant difference was found in time spent in the target quadrant or in the opposite quadrant among groups (*n* = 10 for S and R group, *n* = 15 for M group, *P* > 0.05; Figures 1F,G). However, time spent in the area around the target was lower in the S group (10.4 ± 7.8 s, *n* = 10) as compared to the M group (18.8 ± 9.5 s, *n* = 15, *P* = 0.020) or R group (18.6 ± 7.3 s, *n* = 10, *P* = 0.037; Figure 1H).

### Mitochondrial Bioenergetics Is Lower in the S Group

**Figure 2:**
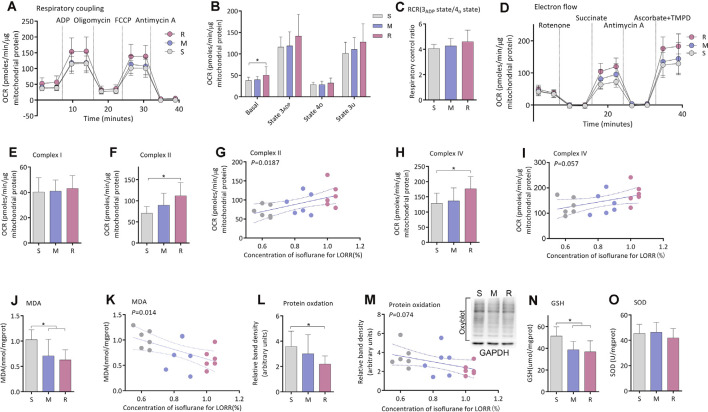
Mitochondrial respiration and brain tissue oxidation measurement. **(A)** The OCR curve of the respiratory coupling experiment (*n* = 6). **(B)** The OCR of Basal, State 3_ADP_, State 4_O_, and State 3_U_ respiration (*n* = 6), *P < 0.05 vs. S group by one-way ANOVA. **(C)** The RCR of 3_ADP_state/4_o_state (*n* = 6), *P* > 0.05 by one-way ANOVA. **(D)** The OCR curve of the electron flow experiment (*n* = 6). **(E)** Complex I respiration (*n* = 6, *P* > 0.05 by one-way ANOVA). **(F)** Complex II respiration (*n* = 6, *P < 0.05 vs. S group by one-way ANOVA). **(G)** Correlation of LORR concentration and complex II respiration (*n* = 6, *r* = 0.548, *P* < 0.05 by Pearson correlation analysis). **(H)** Complex IV respiration (*n* = 6, *P < 0.05 vs. S group by one-way ANOVA). **(I)** Correlation of LORR concentration and complex IV respiration (*n* = 6, *r* = 0.456, *P* < 0.05 by Pearson correlation analysis. **(J)** MDA level in the frontoparietal cortex (*n* = 6, *P < 0.05 vs. S group by one-way ANOVA). **(K)** Correlation of LORR concentration and brain MDA level (*n* = 6, r = –0.570, *P* < 0.05 by Pearson correlation analysis). **(L)** Protein oxidation in the frontoparietal cortex as detected by determining levels of protein carbonylation (*n* = 6, *P < 0.05 vs. S group by one-way ANOVA). **(M)** Correlation of LORR concentration and brain protein oxidation level (*n* = 6, r = –0.431, *P* > 0.05 by Pearson correlation analysis). **(N)** GSH concentration in the frontoparietal cortex (*n* = 6, *P < 0.05 vs. S group by one-way ANOVA). (**O)** SOD level in the frontoparietal cortex (*n* = 6, *P* > 0.05 by one-way ANOVA). S, sensitive group; R, resistant group; M, medium group; LORR, loss of righting reflex; OCR, Oxygen consumption rate; FCCP, carbonyl cyanide 4-(trifluoromethoxy) phenylhydrazone; RCR, respiration control ratio; MDA, malondialdehyde; SOD, superoxide dismutase; GSH, glutathione. Data are presented as mean ± SD.

The coupling assay was employed to detect the degree of coupling between the electron transport chain and oxidative phosphorylation machinery (Figure 2A). The oxygen consumption rate (OCR) of mitochondria was lower in the S group than the R group for basal respiration (37.8 ± 7.9 vs. 54.8 ± 18.9 pmols/min/μg mitochondrial protein, *n* = 6, *P* = 0.035; Figure 2B) when succinate was used as substrate. No difference was found in state 3_ADP_ (*n* = 6, *P* = 0.156; Figure 2B), state 4o (*n* = 6, *P* = 0.469; Figure 2B), or state 3u (*n* = 6, *P* = 0.140; Figure 2B). The respiration control ratio (RCR) calculated by the ratio of state 3_ADP_ to 4o respiration was also similar among the three groups (*n* = 6, *P* = 0.401; Figure 2C).

The electron flow test was used to determine the function of each complex of the electron transport chain (Figure 2D). No difference was found in Complex I respiration among three groups (*n* = 6, *P* = 0.880; Figure 2E). Activity of Complex II in the S group (70.4 16.1 pmols/min/mg mitochondrial protein, *n* = 6) was significantly decreased compared to that of the *R* group (111.8 31.3 pmols/min/mg mitochondrial protein, *n* = 6, *P* = 0.015; Figure 2F. The activity of complex II was positively correlated with the concentration of isoflurane that inducing LORR in mice (*r* = 0.548, *P* = 0.0187; Figure 2G). Complex IV respiration was also lower in the S group (128.2 ± 33.8 pmols/min/μg mitochondrial protein, *n* = 6) than that of the R group (176.4 ± 40.0 pmols/min/μg mitochondrial protein, *n* = 6, *P* = 0.049; Figure 2H). The activities of complex IV (*r* = 0.456, *P* = 0.057; Figure 2I) were positively, albeit not significantly, correlated with the concentration of isoflurane that inducing LORR in mice.

### Oxidative Level of Frontoparietal Cortex Is Higher in S Group

The MDA level of mice frontoparietal cortex was significantly higher in the S group (1.0 ± 0.2 nmol/mg protein, *n* = 6) than that of the R group (0.6 ± 0.2 nmol/mg protein, *n* = 6, *P* = 0.014; Figure 2J). The MDA level was negatively correlated with the concentration of isoflurane required for LORR in mice (*r*0 = −0.570, *P* = 0.014; Figure 2K). The OxyBlot value in the frontoparietal cortex was higher in the S group (3.6 ± 1.2, *n* = 6) than in the R group (2.2 ± 0.6, *n* = 6, *P* = 0.034; Figure 2L). The protein oxidation level was negatively, albeit not significantly, correlated with the concentration of isoflurane required for LORR in mice (*r* = −0.431, *P* = 0.074; Figure 2M). Compared with the R group, the GSH level of the frontoparietal cortex was also significantly higher in the S group than in the R group (51.3 ± 8.5 μmol/mg protein vs. 38.7 ± 7.6, *n* = 6, *P* = 0.012; Figure 2N). However, no difference was found in SOD activity between groups (*P* = 0.565; Figure 2O).

### Oxidation Induced by Iron/Ascorbate Depresses Mitochondrial Respiration

**Figure 3:**
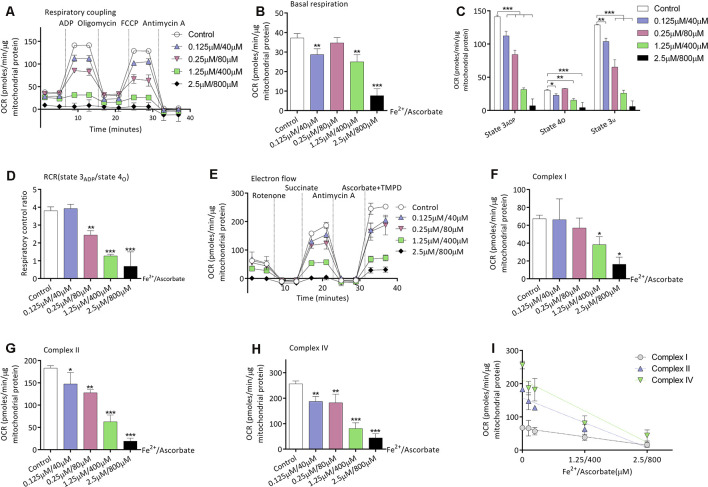
Effects of peroxidation induced by iron/ascorbate on mitochondrial respiration in young adult mice. **(A)** The OCR curve of mitochondria treated with a series of concentrations of iron/ascorbate by respiratory coupling experiment (*n* = 3). **(B)** Effects of peroxidation on basal respiration (*n* = 3, **P < 0.01 and ***P < 0.001 vs. control by one-way ANOVA). **(C)** Effects of peroxidation on State 3_ADP_, State 4_O_, and State 3_U_ respiration (*n* = 3, *P < 0.05, **P < 0.01, and ***P < 0.001 vs. control by one-way ANOVA). **(D)** Effects of peroxidation on RCR (*n* = 3, **P < 0.01 and ***P < 0.001 vs. control by one-way ANOVA). **(E)** The OCR curve of mitochondria treated with a series of concentrations of iron/ascorbate by electron flow experiment (*n* = 3). **(F)** Effects of peroxidation on complex I respiration (*n* = 3, *P < 0.05 vs. control by one-way ANOVA). **(G)** Effects of peroxidation on complex II respiration (*n* = 3, *P < 0.05, **P < 0.01, and ***P < 0.001 vs. control by one-way ANOVA). **(H)** Effects of peroxidation on complex IV respiration (*n* = 3, **P < 0.01 and ***P < 0.001 vs. control by one-way ANOVA). **(I)** Correlation of iron/ascorbate concentration and complexes respiration. OCR, Oxygen consumption rate; RCR, respiration control ratio. Data are presented as mean ± SD.

Given that mice of the S group exhibited higher oxidative stress and decreased mitochondrial respirations in the brain, we then investigated the relationship between oxidation and mitochondrial respirations. Iron/ascorbate was used to induce peroxidation on the mitochondria to detect its effects on mitochondrial bioenergetics (Cardoso et al., 1999). Figure 3A shows the OCR curve of mitochondria treated with a series of concentration of iron/ascorbate by respiratory coupling experiment. Significant decrease of mitochondrial respiration was found in basal respiration (28.6 ± 3.0 vs. 37.2 ± 2.3 pmols/min/μg mitochondrial protein, *n* = 3, *P* = 0.007; Figure 3B), state 3_ADP_ respiration (112.3 ± 7.1 vs. 141.3 ± 2.3 pmols/min/μg mitochondrial protein, *n* = 3, *P* < 0.001; Figure 3C), state 4o respiration (22.8 ± 2.3 vs. 30.5 ± 1.2 pmols/min/μg mitochondrial protein, *n* = 3, *P* = 0.035; Figure 3C), and state 3u respiration (65.3 ± 11.0 vs. 103.8 ± 5.1 pmols/min/μg mitochondrial protein, *n* = 3, *P* = 0.001; Figure 3C) when mitochondria were incubated with 0.125 μM/40 μM iron/ascorbate. However, no change was found in RCR at that concentration (*P* = 0.713; Figure 3D). Figure 3E shows the OCR curve of mitochondria treated with a series of concentrations of iron/ascorbate by electron flow experiment. In the electron flow test, no difference was found in complex I activity when mitochondria were incubated with iron/ascorbate until its concentration reached 1.25 μM/400 μM (67.4 ± 3.8 vs. 38.2 ± 9.1 pmols/min/μg mitochondrial protein, *P* = 0.02; Figure 3F), while activities of complex II (182.9 ± 5.5 vs. 147.4 ± 25.7 pmols/min/μg mitochondrial protein, *P* = 0.012; Figure 3G) and IV (256.3 ± 11.3 vs. 186.9 ± 20.3 pmols/min/μg mitochondrial protein, *P* = 0.003; Figure 3H) were significantly decreased since the iron/ascorbate concentration reached 0.125 μM/40 μM. These results indicate that oxidation can depress the activity of cortex mitochondrial respirations of which Complex II and Complex IV might be more sensitive than Complex I (Figure 3I).

### Malonate Enhances Isoflurane-Induced LORR

**Figure 4:**
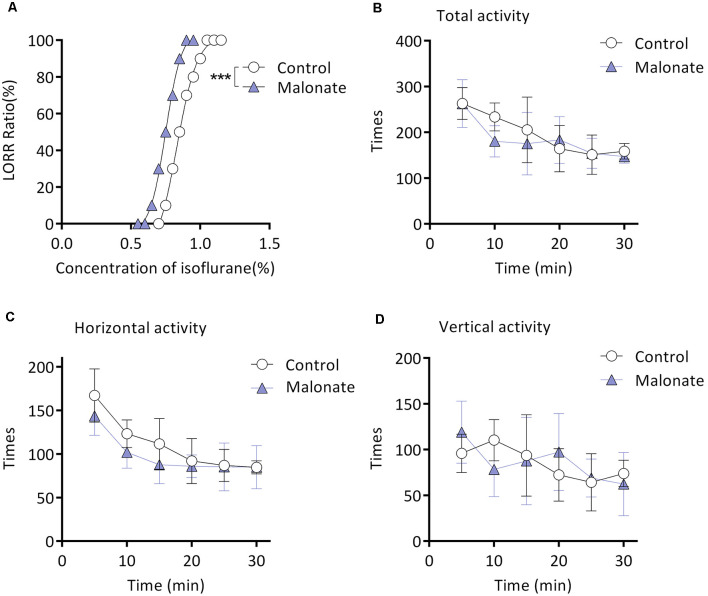
Malonate enhanced isoflurane-induced LORR without affecting the locomotor activities of the aging mice from M group. **(A)** Malonate enhanced isoflurane-induced LORR (*n* = 10, ***P < 0.0001 vs. control). **(B)** The total activity of mice treated with malonate by autonomic activity test (*n* = 8, *P* > 0.05 by two-way ANOVA with repeated measures). **(C)** The horizontal activity of mice (*n* = 8, *P* > 0.05 by two-way ANOVA with repeated measures). **(D)** The vertical activity of mice (*n* = 8, *P* > 0.05 by two-way ANOVA with repeated measures). LORR, loss of righting reflex. Data are presented as mean ± SD.

Twenty mice from the M group were randomly selected for this experiment part. Malonate was served as a competitive inhibitor of Complex II (Wojtovich and Brookes, 2008). MAC_LORR_ was 0.83 (0.81–0.86) % (*n* = 10; Figure 4A) after injection of saline. After preinjection of malonate, MAC_LORR_ decreased to 0.75 (0.73–0.77) % (*n* = 10, *P* < 0.001; Figure 4A). Malonate, at the same dose (375 mg/kg, intraperitoneal injection), did not affect overall activities of the mice (*P* > 0.05; Figure 4B), including number of horizontal (Figure 4C) and vertical movements (Figure 4D). These results indicate that malonate was capable of enhancing the isoflurane-induced LORR and the enhancement might not result from decreased overall activities.

### Ozone Exposure Can Enhance Isoflurane-Induced LORR, Impair Mitochondrial Function, and Exacerbate the Oxidation of Lipid and Protein

**Figure 5:**
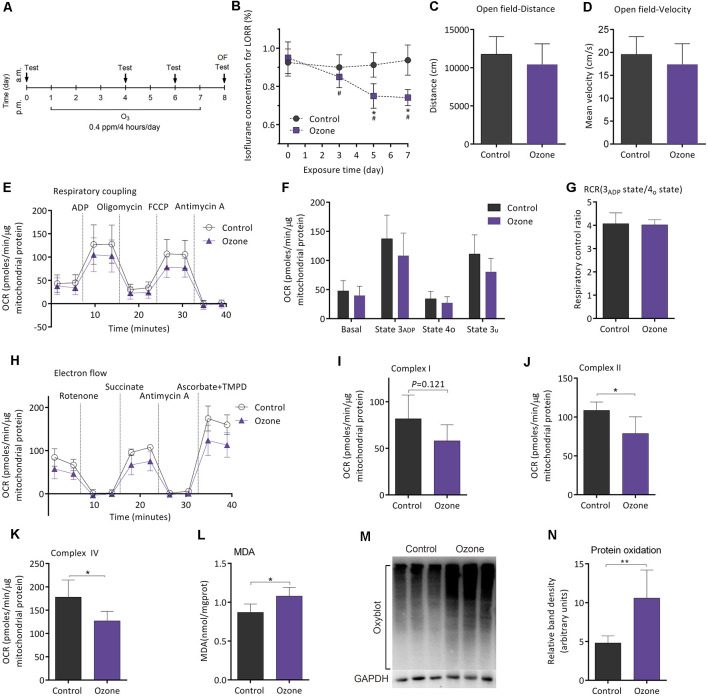
Ozone peroxidation enhanced isoflurane-induced LORR, impaired mitochondrial function, and exacerbated the lipid and protein oxidation in young adult mice. **(A)** The flowchart of ozone exposure. **(B)** Ozone peroxidation gradually enhanced isoflurane-induced LORR (*n* = 12, *P < 0.05 vs. baseline and ^#^*P* < 0.05 vs. control by two-way ANOVA with repeated measures). **(C)** Ozone peroxidation produced no influence in the crossing distance of mice by open field (*n* = 8 for control group and *n* = 12 for ozone group, *P* > 0.05 by two-tailed unpaired student’s *t*-test). **(D)** Ozone peroxidation produced no influence in the mean velocity of mice by the open-field test (*n* = 8 for control group and *n* = 12 for ozone group, *P* > 0.05 by two-tailed unpaired student’s *t*-test). **(E)** The OCR curve of the respiratory coupling experiment (*n* = 5). **(F)** Effects of ozone exposure on the respiratory coupling experiment (*n* = 5, *P* > 0.05 by one-way ANOVA). **(G)** Effects of ozone exposure on the respiratory control ratio (*n* = 5, *P* > 0.05 by one-way ANOVA). **(H)** The OCR curve of the electron flow experiment (*n* = 5). **(I)** Effects of ozone exposure on complex I respiration (*n* = 5, *P* > 0.05 by one-way ANOVA). **(J)** Effects of ozone exposure on complex II respiration (*n* = 5, *P < 0.05 by one-way ANOVA). **(K)** Effects of ozone exposure on complex IV respiration (*n* = 5, *P < 0.05 by one-way ANOVA). **(L)** Effects of ozone exposure on the frontoparietal cortical MDA level (*n* = 5, *P < 0.05 by one-way ANOVA). **(M,N)** Effects of ozone exposure on frontoparietal cortical protein carbonylation (*n* = 5, **P < 0.01 by one-way ANOVA). OCR, oxygen consumption rate; RCR, respiration control ratio; MDA, malondialdehyde; LORR, loss of righting reflex. Data are presented as mean ± SD.

Figure 5A shows the flowchart of ozone exposure experiment. Compared to baseline, MAC_LORR_ was gradually decreased after ozone exposure (*n* = 12, *P* < 0.001; Figure 5B). In control mice (air exposure), MAC_LORR_ was not changed throughout (*n* = 8, *P* = 0.73; Figure 5B). The locomotor activity tested by open field showed no difference in distance (*P* = 0.27; Figure 5C) and mean velocity (*P* = 0.27; Figure 5D) between ozone exposure mice and the control mice. In the mitochondrial respiratory coupling experiment (Figures 5E,F), basal respiration (36.1 ± 16.2 vs. 44.1 ± 17.5 pmols/min/μg mitochondrial protein, *n* = 5, *P* = 0.475), state 3_ADP_ (103.6 ± 35.3 vs. 127.5 ± 41.5 pmols/min/μg mitochondrial protein, *P* = 0.356), state 4o (23.4 ± 12.4 vs. 32.1 ± 12.1 pmols/min/μg mitochondrial protein*, P* = 0.295), and state 3u (77.55 ± 21.1 vs. 106.3 ± 30.4 pmols/min/μg mitochondrial protein, *P* = 0.121) were slightly inhibited by ozone exposure but without statistical significance. The RCR was not significantly affected (5.2 ± 1.2 vs. 4.0 ± 0.2, *n* = 5, *P* = 0.351; Figure 5G). For mitochondrial complex analyses (Figure 5H), activities of complex II (78.8 ± 21.5 vs. 108.6 ± 10.7 pmols/min/μg mitochondrial protein, *P* = 0.024; Figure 5J) and complex IV (127.2 ± 20.4 vs. 178.1 ± 36.6 pmols/min/μg mitochondrial protein, *P* = 0.018; Figure 5K) were significantly decreased by ozone exposure, while no difference was found in complex I activity between the two groups (58.0 ± 17.3 vs. 81.8 ± 25.3 pmols/min/μg mitochondrial protein, *P* = 0.121; Figure 5I). The oxidative effects of ozone exposure on frontoparietal cortical tissue were verified by MDA and Oxyblot test. After ozone exposure, levels of MDA (1.1 ± 0.1 vs. 0.8 ± 0.1 nmol/mg protein, *n* = 5, *P* = 0.018; Figure 5L) and protein carbonylation (10.6 ± 3.6 vs. 4.8 ± 0.9, *n* = 5, *P* = 0.009; Figures 5M,N) were significantly elevated.

### Idebenone Restores Mitochondrial Function and Reduces the Isoflurane Sensitivity in Aging Mice

**Figure 6:**
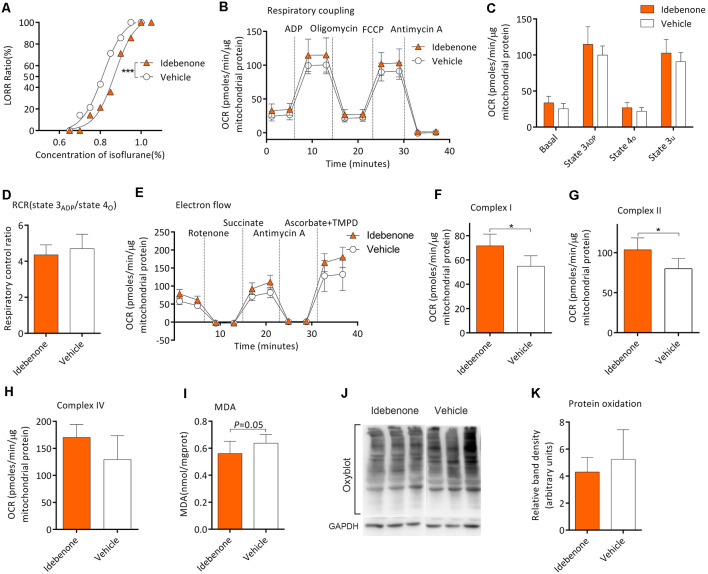
Idebenone reduced isoflurane sensitivity, restored mitochondrial bioenergetics, and alleviated MDA and protein oxidation levels. **(A)** Idebenone increased isoflurane-induced LORR (*n* = 14, ***P < 0.001). **(B)** The OCR curve of respiratory coupling experiment (*n* = 5). **(C)** Effects of idebenone on the respiratory coupling experiment (*n* = 5, *P* > 0.05 by one-way ANOVA). **(D)** Effects of idebenone on RCR (*n* = 5, *P* > 0.05 by one-way ANOVA). **(E)** The OCR curve of electron flow experiment (*n* = 5). **(F)** Effects of idebenone on complex I respiration (*n* = 5, *P < 0.05 by one-way ANOVA). **(G)** Effects of idebenone on complex II respiration (*n* = 5, *P < 0.05 by one-way ANOVA). **(H)** Effects of idebenone on complex IV respiration (*n* = 5, *P* = 0.107 by one-way ANOVA). **(I)** Effects of idebenone on the frontoparietal cortical MDA level (*n* = 9, *P* = 0.05 by one-way ANOVA). **(J,K)** Effects of idebenone on frontoparietal cortical protein carbonylation (*n* = 12, *P* = 0.199 by one-way ANOVA). OCR, oxygen consumption rate; RCR, respiration control ratio; MDA, malondialdehyde; LORR, loss of righting reflex. Data are presented as mean ± SD.

Twenty-eight mice from the M group were randomly selected for this experiment part. After gavage for 60 days, MAC_LORR_ was 0.81 (0.79–0.84) % (*n* = 14; Figure 6A) and 0.87 (0.85–0.89) % (*n* = 14, *P* < 0.001; Figure 6A) in the vehicle group and idebenone group, respectively. In the respiratory coupling experiment (Figures 6B,C), basal respiration (25.6 ± 7.2 vs. 26.9 ± 7.5 pmols/min/μg mitochondrial protein, *n* = 5, *P* = 0.163), state 3_ADP_ (99.9 ± 12.7 vs. 114.9 ± 24.6 pmols/min/μg mitochondrial protein, *P* = 0.263), state 4o (21.9 ± 5.3 vs. 26.9 ± 7.5 pmols/min/μg mitochondrial protein, *P* = 0.256), and state 3u (90.7 ± 12.7 vs. 102.7 ± 18.8 pmols/min/μg mitochondrial protein*, P* = 0.271) were slightly elevated in idebenone group but without statistical significance as compared to the vehicle group. The RCR was also slightly decreased in the idebenone group but without statistical significance as compared to vehicle group (4.7 ± 0.8 vs. 4.3 ± 0.6, *n* = 5, *P* = 0.455; Figure 6D). In the electron flow experiment, complex I respiration (54.9 ± 8.7 vs. 71.6 ± 9.7 pmols/min/μg mitochondrial protein, *n* = 5, *P* = 0.02; Figures 6E,F) and complex II respiration (79.9 ± 12.9 vs. 103.7 ± 14.9 pmols/min/μg mitochondrial protein, *n* = 5, *P* = 0.028; Figure 6G) were significantly elevated by idebenone treatment. However, Complex IV respiration (129.5 ± 44.3 vs. 170.3 ± 23.9 pmols/min/μg mitochondrial protein, *n* = 5, *P* = 0.107; Figure 6H) was only slightly improved but without statistical significance in the idebenone group as compared to the vehicle group. Nine samples and twelve samples from the frontoparietal cortex were randomly picked for MDA and Oxyblot analysis, respectively. The MDA level was decreased (0.64 ± 0.06 vs. 0.56 ± 0.09 nmol/mg protein, *n* = 9, *P* = 0.05; Figure 6I), while the OxyBlot value was slightly lower (5.24 ± 2.20 vs. 4.30 ± 1.07 nmol/mg protein, *n* = 12, *P* = 0.199; Figures 6J,K) but without statistical significance after idebenone treatment. These results indicated that idebenone was capable of restoring the mitochondrial function, alleviating the lipid/protein oxidation, and decreasing the sensitivity to isoflurane in aging mice.

## Discussion

In the present study, we demonstrated that the function of mitochondrial complexes and oxidative status of the frontoparietal cortex may be related to the enhanced sensitivity to isoflurane in aging mice. The aging mice with higher sensitivity to isoflurane exhibited decreased respirations of mitochondrial complex II and complex IV and elevated oxidation stress levels of the frontoparietal cortex.

In clinical patients, the MAC of volatile anesthetics decreases with aging (Chemali et al., 2015). The depth of general anesthesia might predict postoperative outcomes (Sessler et al., 2012; Willingham et al., 2015; Zimin et al., 2016). Oxidative stress and impaired mitochondrial function are core factors involved in aging, which might also lead individuals to be more vulnerable to various diseases (Kauppila et al., 2017). In this study, we found that overall exercise capacity together with learning and memory function was also reduced in the aging mice with higher sensitivity to isoflurane. These results suggest that high sensitivity to isoflurane might represent a status with progressive loss of physiological integrity.

Several studies showed that general anesthetics, such as propofol (Mansouri et al., 2019) and xenon (Baumert, 2009), might exhibit gender-dependent effects. Cesarovic et al. (2010) found that the female mice might have higher MAC value than the male. Besides, lots of studies found that sex differences affected brain morphology and physiology during both development and aging (Evola et al., 2018; Goyal et al., 2019). Decline of estradiol in the aged female mice corresponded with significant changes in various protein concentrations throughout the brain, especially in the frontal cortex and hippocampus. Notably, many of these changes occurred within mitochondria (Evola et al., 2018). Therefore, we selected male mice only in this present study to minimize potential confounds.

Several brain regions including frontoparietal cortex, dorsal anterior cingulate gyrus, and brain stem were reported to be involved in hypnotic effects of volatile anesthetics (Speigel et al., 2017). In this study, we investigated whether there was any intrinsic link among mitochondrial function, oxidative stress of the frontoparietal cortex, and anesthetic sensitivity in aging mice. Previous studies showed that lipid and protein peroxidation were common markers of oxidative stress levels. MDA are breakdown products of fatty acid peroxidation while protein carbonylation reflects the levels of protein oxidation (Jacob et al., 2013). In this study, MDA and protein carbonylation levels were found elevated in the mice from the S group. Thus, the mice with higher sensitivity to isoflurane might have higher levels of oxidative stress. Lipid peroxidation may lead to some adverse effects on membrane physiology. First, membrane peroxidation is likely to become rigid and lose selective permeability (Sharma et al., 1993). Lipid peroxidation can modify functions of membrane enzymes, receptors (Goel et al., 1994), and ion channels (Blanc et al., 1998). Additionally, oxidative stress has been proved to compromise both neuronal excitability and the capacity of generating of action potentials (Pardillo-Diaz et al., 2015). These results are in agreement with the fact that oxidation can enhance the sedative effects of diazepam and xylazine (Mousa and Mohammad, 2012). Here, we found that ozone exposure elevated the oxidation levels of frontoparietal cortical tissue (lipid and protein) and increased the isoflurane sensitivity *in vivo*. Therefore, the increased isoflurane sensitivity might be related to peroxidation status of the frontoparietal cortex.

Because decreased activities of mitochondrial complexes and elevated levels of oxidative stress coexisted in the mice with higher isoflurane sensitivity, we next investigate whether oxidative stress can affect the activities of mitochondrial complexes. Our results indicated that the basal respiration, state 3_ADP_, state 4o, and state 3u respirations were all impaired along with the elevated levels of oxidation increased *in vitro*. Besides, we also found a downward trend in these aspects after ozone exposure *in vivo*. Interestingly, activities of complex II and complex IV are more sensitive to oxidation induced by ozone exposure *in vivo* or oxidation induced by iron/ascorbate treatment *in vitro* as compared to complex I. These results were similar to the studies by Van der Zee et al. (1987), Cardoso et al. (1999) and Valdez et al. (2019). We speculated that the effects of oxidative stress on mitochondrial coupling bioenergetics and complex activities would be more pronounced with the higher concentration and longer duration of ozone exposure. The levels of oxidative stress were elevated accompanied with the reduction of mitochondrial complex activities in isoflurane-sensitive mice, suggesting that the oxidative stress might at least partly mediate the changes of mitochondrial complexes respirations in isoflurane sensitive mice.

Mitochondria, of central importance for multiple cellular processes such as ATP production, apoptosis, and β-oxidation of fatty acids, are believed to be responsible for the production of free radicals. Previous studies reported that defects of complex I of mitochondria contributed to increased anesthesia sensitivity in animals (Guo et al., 2017; Zimin et al., 2018). In the present study, decreased respirations of complex II and complex IV were observed in the mice with higher isoflurane sensitivity. Additionally, we found that malonate, a competitive inhibitor of succinate dehydrogenases (SDH), enhanced isoflurane-induced LORR, indicating that impaired function of complex II might enhance isoflurane sensitivity.

Although no difference was found in the activity of complex I between S group and R group, the effects of complex I on anesthetics sensitivity should not be ignored because defects of complex I can increase anesthetics sensitivity (Quintana et al., 2012). Similarly, single-cell transcriptomic analysis indicates that the expression of the genes related to mitochondrial complex I (mt-Nd1, mt-Nd4) is significantly decreased with aging (Ximerakis et al., 2019). However, in this study, effects of complex I seems not be associated with isoflurane sensitivity in aging mice. It is possible that the respiration of complex I might decrease with similar degrees in all the aging mice. Of note, the comparisons between young mice and aging mice or among aging mice are different. Among aging mice, differential oxidative status and impaired functions of complex II and complex IV can be more related to isoflurane hypersensitivity. Interestingly, change of functions of mitochondrial complexes may be not linear with aging (Stauch et al., 2014). Quantitative proteomics reveals that the subunit expression of the electron transport chain decreases from the age of 5 months to 12 months in mice while it increases from age of 12 months to 24 months, suggesting that a dynamic change of the mitochondrial proteome may serve as a compensatory mechanism to age-related decline of the electron transport chain (Stauch et al., 2014).

Ozone exposure is commonly used to induce oxidative stress characterized by lipid peroxidation *in vivo* (Guevara-Guzman et al., 2009). Our result showed that ozone exposure induced lipid and protein oxidation, impaired mitochondrial function, and enhanced isoflurane-induced LORR *in vivo*, suggesting that elevated oxidative levels impaired the mitochondrial function, which might together contribute to hypersensitivity to isoflurane *in vivo*. Idebenone, an analog of coenzyme Q10, a free radical scavenger capable of crossing the blood brain barrier, has been proved to rescue the SDH activity and reduce peroxidation (Rauchova et al., 2012). After treatment with idebenone, the levels of lipid oxidation were alleviated and mitochondrial complex I and II respiration was restored in aging mice. Additionally, the concentration of isoflurane that is required for LORR was also increased after idebenone treatment. Together with the results above, we speculated that the levels of oxidative stress in the frontoparietal cortex might partly contribute to the mitochondrial bioenergetics polymorphism and the sensitivity to isoflurane.

There are several limitations in this study. First, there is no highly selective complex II blocker; hence, a genetically engineered animal model with a certain complex II defect may be needed to further confirm the role of complex II in isoflurane sensitivity. Meanwhile, because complex IV inhibitors are lethal, we did not test the contribution of complex IV in sensitivity to isoflurane *in vivo*. Second, we did not explore whether mitochondrial complexes contribute to isoflurane sensitivity between young and aging mice. Finally, this present study was performed only in male mice; therefore, the conclusions should be treated cautiously to the female.

In conclusion, we indicate that the aging mice with hypersensitivity to isoflurane exhibit reduced respirations of mitochondrial complex II and complex IV and elevated oxidative levels of the frontoparietal cortex. Oxidation can depress mitochondrial respirations of which complex II and complex IV are more sensitive than complex I. Malonate or ozone exposure can enhance isoflurane sensitivity without disturbing overall activity. On the other hand, antioxidative therapy by idebenone treatment alleviated the oxidative stress levels, restored the mitochondrial complexes respiration, and elevated the concentration of isoflurane that is required for LORR. Therefore, higher oxidative stress status and impaired mitochondrial functions in the frontoparietal cortex may contribute to the hypersensitivity to isoflurane in aging mice.

## Data Availability Statement

The raw data supporting the conclusions of this article will be made available by the authors, without undue reservation.

## Ethics Statement

The animal study was reviewed and approved by the Institutional Animal Care and Use Committee of West China Hospital (Sichuan University, Chengdu, China).

## Author Contributions

CG, DZ, and WO: draft of the manuscript, study concept and design, and acquisition of the data and analysis. MO, PL, DL, WZ, and JL: acquisition of the data and interpretation of the data. TZ and CZ: revision of the manuscript, study concept and design, and study supervision. All authors contributed to the article and approved the submitted version.

## Conflict of Interest

The authors declare that the research was conducted in the absence of any commercial or financial relationships that could be construed as a potential conflict of interest.
